# Production and cross-sectional characterization of aligned co-electrospun hollow microfibrous bulk assemblies

**DOI:** 10.1016/j.matchar.2015.09.010

**Published:** 2015-11

**Authors:** Feng-Lei Zhou, Geoff J.M. Parker, Stephen J. Eichhorn, Penny L. Hubbard Cristinacce

**Affiliations:** a Centre for Imaging Sciences, The University of Manchester, Manchester M13 9PT, UK; b The School of Materials, The University of Manchester, Manchester M13 9PL, UK; c CRUK-EPSRC Cancer Imaging Centre in Cambridge and Manchester, UK; d College of Engineering, Mathematics and Physical Sciences, University of Exeter, Exeter EX4 4QF, UK; e School of Psychological Sciences, University of Manchester, Manchester M13 9PT, UK

**Keywords:** Co-electrospinning, Hollow microfibers, SEM, Cross-section, Production time

## Abstract

The development of co-electrospun (co-ES) hollow microfibrous assemblies of an appreciable thickness is critical for many practical applications, including filtration membranes and tissue-mimicking scaffolds. In this study, thick uniaxially aligned hollow microfibrous assemblies forming fiber bundles and strips were prepared by co-ES of polycaprolactone (PCL) and polyethylene oxide (PEO) as shell and core materials, respectively. Hollow microfiber bundles were deposited on a fixed rotating disc, which resulted in non-controllable cross-sectional shapes on a macroscopic scale. In comparison, fiber strips were produced with tuneable thickness and width by additionally employing an *x*–*y* translation stage in co-ES. Scanning electron microscopy (SEM) images of cross-sections of fiber assemblies were analyzed to investigate the effects of production time (from 0.5 h to 12 h), core flow rate (from 0.8 mL/h to 2.0 mL/h) and/or translation speed (from 0.2 mm/s to 5 mm/s) on the pores and porosity. We observed significant changes in pore size and shape with core flow rate but the influence of production time varied; five strips produced under the same conditions had reasonably good size and porosity reproducibility; pore sizes didn't vary significantly from strip bottom to surface, although the porosity gradually decreased and then returned to the initial level.

## Highlights

• Hollow microfibrous assemblies based on co-electrospinning are demonstrated.
• The thickness and width of co-electrospun strips were controllable.
• Cross-sections of fibres had non-normally distributed pore sizes and shapes.
• Cross-sections were significantly influenced by production time and flow rate.
• Co-electrospun strips had reasonably good reproducible cross-sections.

## Graphical abstract

**Figure:**
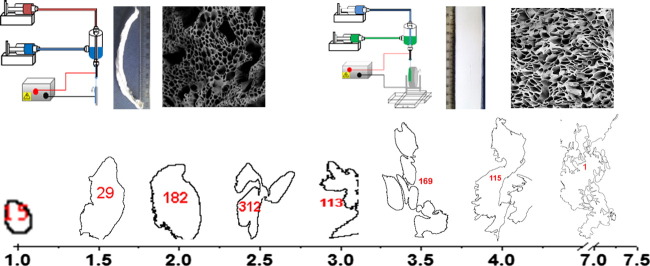

## 1 Introduction

Nanofibers produced by electrospinning often have diameters of a few orders of magnitude smaller than those in conventional hollow fiber membranes [1]. Electrospun (ES) nanofibers for membrane applications [2] are also solid and are often produced in the form of nonwoven meshes. Recently core/shell [3] or hollow [4] polymeric fibers having diameters of only a few microns or less have been produced by coaxial electrospinning (co-ES) for different applications, ranging from superhydrophobic and oleophobic fibers [5], self-healing coatings [6], self-cleaning textiles [7], multi-agent or drug delivery [8], [9], and liquid filtration media [10], [11]. In these applications, co-ES microfibrous assemblies often have to be of millimeter thickness. This amount of material can take a lab-scale setup a few hours or more to produce, due to low mass throughputs [11], [12].

The production and reproducibility of millimeter-thick co-ES microfibrous structures has scarcely been explored. There have been only a few reports on aligned thick co-ES hollow microfiber membranes, which have application as, for example, anisotropic nerve and vascular scaffolds [13], ultrafiltration membranes [10] and biomimetic fibrous membranes [14], [15], which could be processed into woven or knitted protective fabrics where hollow microfiber membranes are often sandwiched between two layers of fabrics [16]. Recently we have shown that co-ES microfibers can be used to mimic cell structures and provide materials that can be imaged using diffusion magnetic resonance imaging (MRI) to provide signals that resemble brain tissue to provide MRI scanning performance information [14], [17]. The growth in the number of applications for bulk samples of co-ES microfibers indicates that a study is needed to optimize co-ES process parameters that determine the formation of structurally reproducible mm-thick microfibrous membranes.

In order to obtain fibrous assemblies with sufficient thickness for easy handling and for applications such as those described above, ES [18], [19], [20] and co-ES processes [14], [21], [22], [23] have to be operated for long periods of time. To date, there have been no reports on the effect of production times on the structure of co-ES microfibers, though a few studies have been published on the effect of production time on fiber alignment [18], drug release [24], porosity and mechanical properties of ES nanofiber meshes [19], [20]. In the aforementioned report of aligned co-ES fiber bundles used to mimic brain white matter structures [14], [21], Zhou et al. [14] presented the results of aligned bundles of co-ES hollow microfibers, which were prepared for around 35 min and were used for the validation of diffusion MRI. Aligned co-ES core–shell fibers with tuneable mechanical and chemical properties were also produced for around 45 min by Rao et al. [21] as white matter-mimicking scaffolds for examining migration of cells associated with malignant brain tumors. However, the limited thickness of co-ES microfiber structures poses assembly problems for practical applications. For example, the tissue-mimicking prototypes developed to date have to be composed of several layers of fiber bundles, which is likely to result in the partial loss of structural integrity and performance. A similar problem exists for hollow fiber filtration modules in which sheets of co-ES fibers prepared for 2.5 h still had to be layered or rolled up into a cylinder shape to form a thick enough construct [10], [11]. The low structural integrity of developed filtration modules could compromise the filtration efficiency of co-ES fibers due to the use of epoxy glue to connect separate layers of fibers.

In addition to the production challenges, cross-sectional characterization on the macroscopic and microscopic scale of millimeter-thick co-ES hollow microfiber assemblies also remains challenging. ES fiber diameters [25] and co-ES fiber inner and outer diameters [14], [21], [26] are often measured manually via off-the-shelf software packages, for instance using ImageJ's (http://imagej.nih.gov/ij) line-drawing feature. However, these larger samples may require more automated methods of measurement. The pore size and porosity of ES random fiber meshes can be measured by using the ‘thresholding’ feature of this software [27]. This feature will be adopted here to measure the cross-sectional pore size and porosity of aligned co-ES microfibers.

In the present study, co-ES hollow PCL microfibers were produced in aligned assemblies over longer production times than those previously reported in other studies. Fiber bundles were produced over time scales of between 0.5 and 2 h and fiber strips between 6 and 12 h. The cross-sections of the resultant co-ES microfiber assemblies were cleaved using a freeze fracture technique and microstructural images were acquired using scanning electron microscopy (SEM). SEM images were processed and analyzed using ImageJ to determine the cross-sectional pore sizes and porosities. The effect of core flow rate, production time and translation speed on the cross-sections of co-ES microfibers was also investigated. The cross-sectional reproducibility and variation of the co-ES fiber strips was assessed using SEM from five samples produced using the same experimental conditions. Finally, the effects of production time on sample microstructure were assessed by dividing a cross-sectional image into ten sections corresponding to regions deposited during different periods of the overall production time.

## 2 Experimental

### 2.1 Materials

Polycaprolactone (PCL, number-average molecular weight M_n_ = 70,000–90,000 g/mol) and polyethylene oxide (PEO, with an average viscosity molecular weight M_v_ = 900,000 g/mol) were obtained from Sigma Aldrich (Dorset, UK) and used as received. The solvents chloroform and N,N-dimethyl-formamide (DMF) were also purchased from Sigma Aldrich (Dorset, UK). Deionized water was used to dissolve PEO.

### 2.2 Coaxial electrospinning of hollow microfiber assemblies

**Fig. 1:**
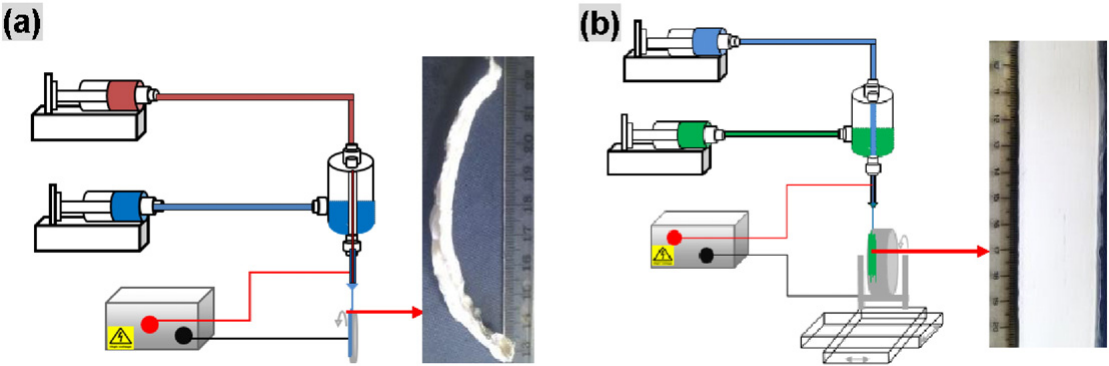
Schematics of co-ES for the production of (a) microfiber bundle on a rotating disc; and (b) microfiber strip on a rotating drum mounted on *x–y* translation stage.

Co-ES was performed on a lab-scale setup (see details in ref. [28]). All experiments were conducted in a fume cupboard under ambient conditions. In a typical procedure for co-ES, 10 wt.% PCL in chloroform/DMF (80/20, wt.%) and 4 wt.% PEO in deionized water were used as the shell and core polymer solutions, respectively. These two polymer solutions were fed at constant flow rates, independently controlled by two syringe pumps. The flow rate for the PCL solution was set at 3 mL/h; for the PEO solution, the flow rate was varied from 0.8 mL/h through 1.0 mL/h to 1.4 mL/h. The applied voltage was set at 9 kV and the working distance between the coaxial spinneret and the fiber collector was 5 cm. To investigate the effect of the production time on cross-sections, PCL hollow microfibers were produced in the form of aligned bundles on a rotating disc over a period between 0.5 h and 2 h. For the reproducibility study, aligned PCL hollow microfiber strips were also produced on a rotating drum mounted on an *x*–*y* stage over a 6 h period using core flow rates of 0.8 mL/h, 1.4 mL/h and 2.0 mL/h. The width of the strips was set at 30 mm and the resultant thickness was 0.5–0.8 mm, as measured by a digital micrometer. A schematic of the production of aligned co-ES hollow microfiber bundles and strips is shown in Fig. 1.

### 2.3 Freeze fracture and SEM characterization

**Fig. 2:**
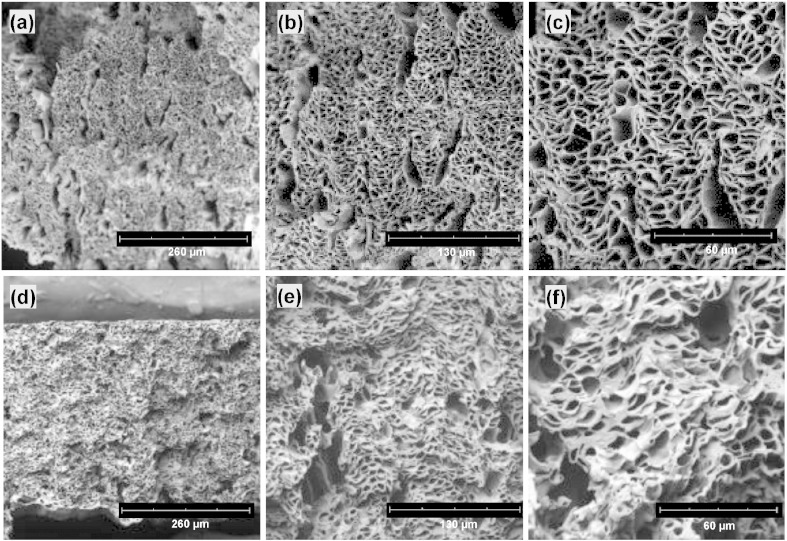
Typical SEM images of the cross-sections of (a–c) one co-ES microfiber bundle and (d–f) one strip with × 500, × 1000, and × 2000 magnifications. Note — the apparent deformation of the ends of the fibers along the plane of the cut of the strips (d–f).

Freeze fracture has been used to prepare cross-sections of hollow fiber polymeric membranes for microfiltration [29] and co-ES microfibers [10] for visualization using SEM. Co-ES fiber bundles/strips were firstly removed from the collector and then immersed in a liquid nitrogen bath until the sample was completely frozen. Sharp scissors and scalpels were then cooled in the bath, and used to quickly cut the fiber bundles and strips in one step. The cross-sections of co-ES fibers were observed using a Phenom G2 pro desktop SEM (Phenom-World). The co-ES fiber specimens were coated with a thin gold film to increase their conductivity. Typical examples of the fractured cross-sections of co-ES microfiber bundles and strips with a common feature of a porous microstructure are shown in Fig. 2a–c and d–f, respectively. SEM images taken with an acceleration voltage of 5 kV and various magnifications were used for further image processing. Cleaving hollow fibers in liquid nitrogen could still result in significant mechanical deformation in some samples (e.g. Fig. 2f). In addition, because of the nature of the method, there is a practical upper limit to the size of the sample that can be prepared (i.e. it has to be no more than a few millimeters thick).

### 2.4 Pore diameter and porosity quantification

**Fig. 3:**
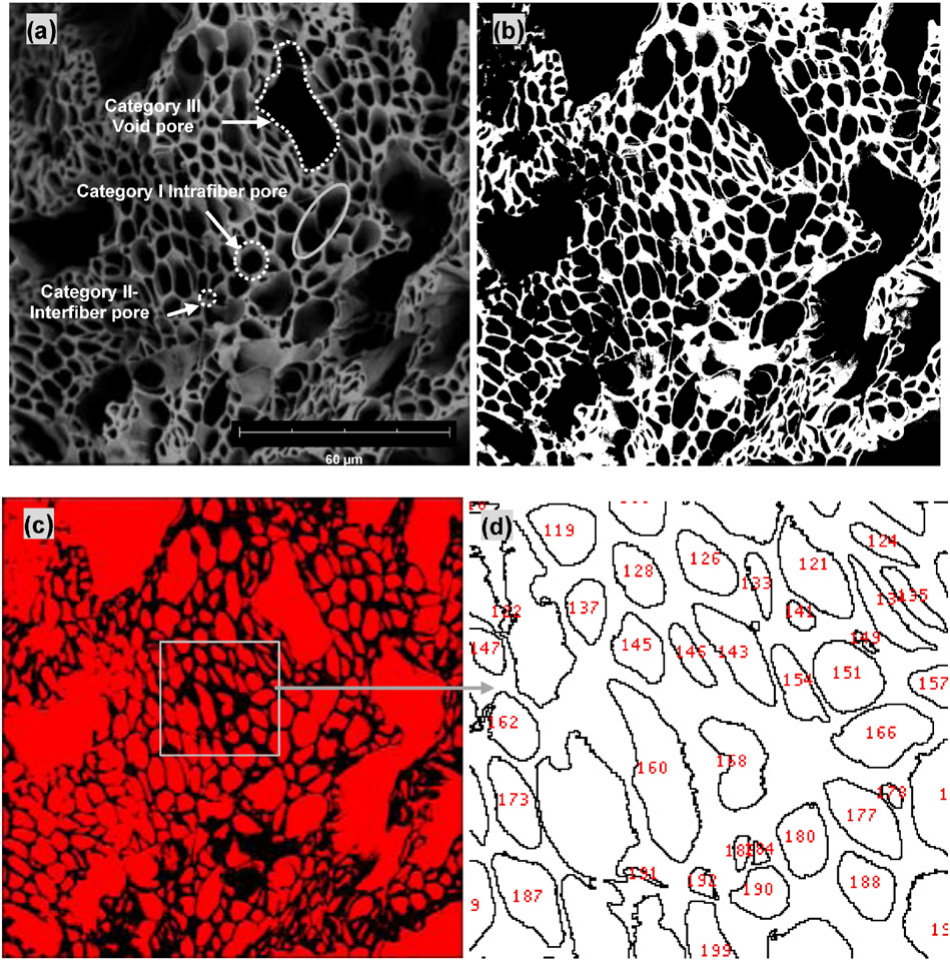
(a) SEM image of a representative co-ES fiber bundle cross-section; (b) the corresponding binary converted image of (a), with fiber walls depicted in white; (c) area porosity (65.7%) indicated in red after image thresholding; (d) a zoomed-in view of the identified pores, denoted by their unique index number in red.

The porous structure is the main geometrical microstructural feature of the cross-sections of co-ES aligned microfiber bundles and strips. Therefore, measurement parameters including pore size and porosity were determined from representative SEM images of these materials. Pores of microfiltration membranes were categorized into two types to define their structures, namely separated and connected, as previously reported [30]. As shown in Fig. 3a, three categories of pores were observed on representative SEM images of the cross-sections of co-ES microfiber bundles: (I) intrafiber pores derived from hollow microfibers, usually well-defined and with diameters from a few microns of individual fibers to tens of microns of two neighbouring fibers that have lost their separating walls (see ellipse in grey); (II) interfiber pores defined by the merged walls of neighbouring hollow microfibers, also well-defined but generally smaller in size than those in category I; (III) void pores, usually having the largest sizes (these can be considered extreme examples of type II pores but justify their separate classification due to the potentially large sizes of these features). Among these pores (Fig. 3a), category I are controllable by co-ES parameters, for example, the core flow rate, whereas other types of pores were formed randomly, independent of the co-ES process, at least under ambient conditions.

A number of ways to characterize pore sizes, including number-averaged, area-averaged, and volume-averaged, have been proposed to calculate an equivalent representative unimodal diameter in filters composed of fibers with a bimodal (or multimodal) fiber diameter distribution [31], [32]. The cross-sections of co-ES fiber assemblies (Figs. 2c, f and 3a) exhibited a wide range of pore sizes from about 1 μm in category II to about 10 μm or larger in categories I and III. Only area-averaged and number-averaged pore sizes could be used for the cross-sections of co-ES fiber bundles/strips, since 2D SEM images cannot be used to get volume-averaged pores.

The cross-sectional porosity of co-ES microfiber bundles and strips was calculated using ImageJ software by converting an SEM image (e.g. Fig. 3a) to a binary image and thresholding. The pore area was then calculated by using the ImageJ ‘Analyse Particles’ feature. Fig. 3b and c show typical binary-converted and thresholded images, respectively. The numbers reported in the zoomed-in inset in Fig. 3d are the sequential index of the identified pores. The percentage of the white or red area relative to the total area in each image was defined as the percent porosity according to the equation:

(1) $$\emptyset =\frac{\sum_{i=1}^{n}A_{i}}{A}$$

where *A*_*i*_ and *A* are the pore area of each *i*^th^ measured pore and total area of the measured image.

The automatic measurements of each pore area (*A*_*i*_) were converted into the corresponding pore diameter (*d*_*i*_) assuming that the cross-section pore is circular:

(2) $$d_{i}=2\sqrt{\frac{A_{i}}{\pi }}$$

where *d*_*i*_ is the diameter of each *i*^th^ measured pore.

Not only the amount of porosity but also the pore geometry, affects the physical properties of co-electrospun hollow fibers, as shown in applications such as brain-mimicking phantoms for diffusion magnetic resonance imaging. In this study, we also introduce a dimensionless parameter (*γ*) [33], indicating pore shape, the ratio of pore perimeter (*P*) to the square root of pore area (*A*), which becomes 1 for a circle:

(3) $$\gamma =\frac{P_{i}}{2\sqrt{\pi A_{i}}}$$

### 2.5 Statistical analysis

All the data were analyzed regarding skewness and kurtosis (SPSS version 22, Chicago, IL, USA) to determine if the data were normally or non-normally distributed. Data with a skewness value close to zero (less than twice its standard error) and a kurtosis value also close to zero were considered normal. The data on pore size and pore shape parameter were not normally distributed and were analyzed using Kruskal–Wallis ANOVA (SPSS). Statistical significance was accepted at p < 0.05.

## 3 Results and discussion

### 3.1 Effect of production time on cross-sections of co-ES fiber bundles

**Fig. 4:**
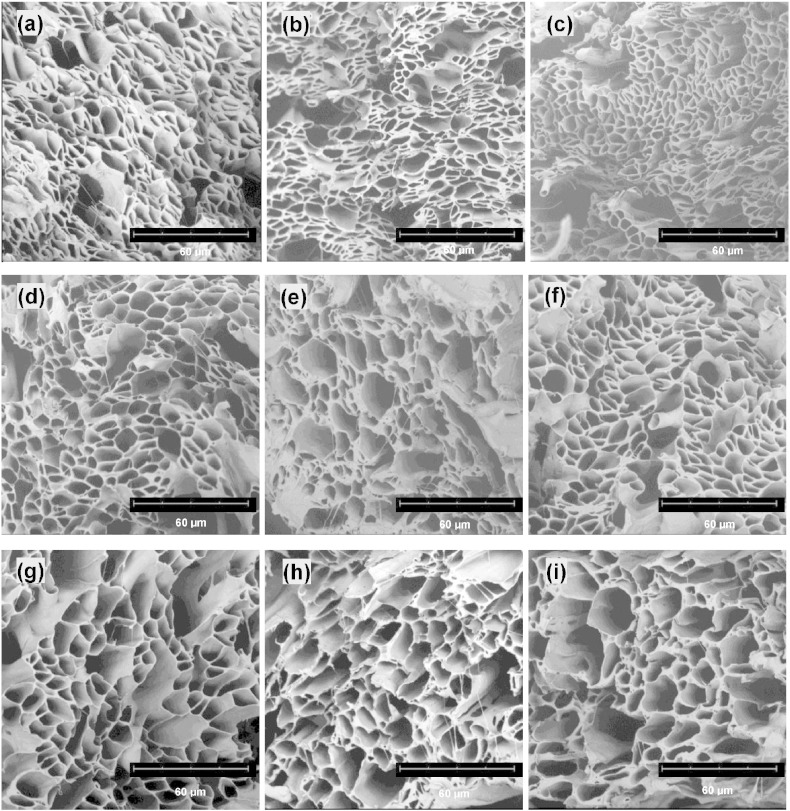
SEM images of cross-sections of co-ES fiber bundles produced at different combinations of core flow rate-production time. (a–c) 0.8 mL/h — 0.5 h, 1 h, 2 h; (d–f) 1.0 mL/h — 0.5 h, 1 h, 2 h; (g–i) 1.4 mL/h — 0.5 h, 1 h, 2 h.

There have been a limited number of studies on the effect of production time on ES nanofibers. [18], [19], [20] For instance, Katta et al. [18] reported that increasing the production time from 5 min to 2.5 h resulted in the loss of fiber alignment in thicker electrospun nylon-6 mats. More recently, it has been reported by Han et al. [24] that an increase in the production time could result in longer and more linear release of hydrophilic drugs encapsulated in ES hydrogel composite fibers. Essalhi et al. [19], [20] found that the production time had no effect on the diameter and surface wettability of ES PVDF fibers produced for 1 to 4 h. However, there was a significant decrease in the pore size and a slight increase in porosity of ES fibrous meshes [19]. In order to investigate the potential effect of production time on porosity, here aligned fiber bundles were produced over different periods of time ranging from 30 min to 2 h. Some representative SEM images of fibers produced using this method are shown in Fig. 4.

Despite the simplicity of the one-step formation of hollow microfibers by co-ES [4], if the “right” combination of applied voltage and core/shell flow rate for a specific core/shell solution pair is not used, this poses a challenge for the formation of mm-scale thickness assemblies and to the stability of the co-ES process. The parameters used in this study were optimized from our previous study [14], which allows a stable operation of the co-ES process over a period of a few hours. It should be noted that the 5 cm working distance used in this study was much smaller than those previously reported, for example, 16–20 cm [4], resulting in a significantly different jet behaviour. Using a 5 cm working distance, fiber deposition was controlled by a linear straight jet, as the bending instability which usually occurs in conventional ES was suppressed [28]. The suppression of the bending instability, however, may lead to fiber accumulation and build-up at one position on the rotating collector, as reported by Ou et al. [13]. As a result, we found that the process was only stable for a limited period of time because the increasing height of fiber layers led to an interruption of the jet. During the production of fiber bundles, it was observed that the maximum operation time that allowed the straight stable jet to deposit on the collector, was around 2 h. Therefore, fiber bundles used to investigate the effect of operation time were produced for up to 2 h.

The cross-sections of resultant fiber bundles were prepared using the freeze fracture method and imaged using SEM. As shown in representative SEM images in Fig. 4, the two dominant characteristic structural features of each bundle produced using times of 0.5 h, 1 h and 2 h are the porous cross sections and the merging of neighbouring hollow microfibers. Clearly the majority of microsized pores were formed by co-ES hollow microfibers. However, most hollow fibers became merged, possibly due to incomplete solvent evaporation and/or parallel alignment of fibers, resulting in a significant decrease in interfiber spacings. There were also relatively large pores (category III) between hollow microfibers, which resulted in poor fiber packing in localized areas within aligned bundles.

**Fig. 5:**
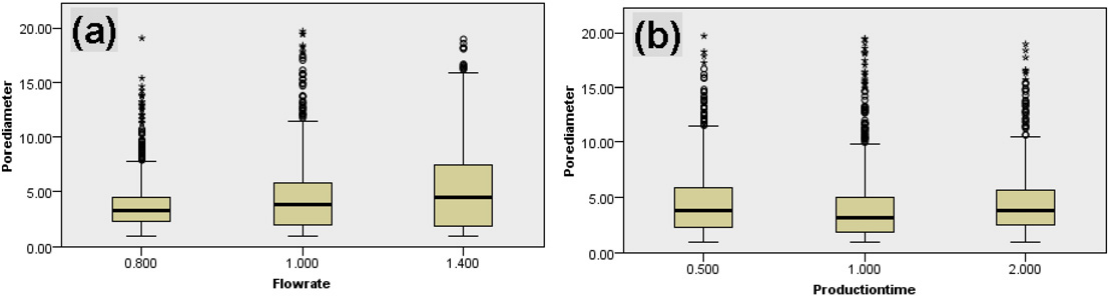
Box-and-whisker plots depicting the dependence of pore size of hollow fiber bundles with flow rate and production time. (a) Core flow rate (0.8 mL/h, 1.0 mL/h and 1.4 mL/h); (b) production time (0.5 h, 1.0 h and 2.0 h). Median values, interquartile ranges (i.q.r.) and 1.5 times the i.q.r (excluding outliers ° and extreme values *) are denoted by horizontal bars, boxes and whiskers respectively. There were statistically significant changes in pore sizes with the increasing core flow rate and production time (Kruskal–Wallis, p < 0.01).

Fig. 5 reports box-and-whisker plots showing the median and interquartile range of pore sizes of fiber bundles produced using the various combinations of production times and core flow rates, and also demonstrating the differences among fiber bundles. Individual plots of pore size show there were wider variations with increasing core flow rate (Fig. 5a); but the variation was less with increasing production time (Fig. 5b). The pore size of fiber bundles increased with core flow rate, but with production time first declined and then increased by a small amount. However, these changes in pore size with both flow rate and production time were significant (Kruskal–Wallis, p < 0.01). Spearman's rho correlation further suggests that there was a significant correlation between flow rate and pore diameter (rho = 0.193; p < 0.01, 2-tailed) but not between production time and pore diameter (rho = 0.002; p = 0.928, 2-tailed). Therefore, the implication is that there is a small amount of variability between co-ES experiments but that for production time this variability is non-linear or unpredictable, whereas with flow rate there is a weak ability to predict an increase in pore size with increasing flow rate. A large number of outliers and extremes were observed for those converted pore sizes from measured pore areas indicating that category III void pores were present in most of the nine fiber strips, which contributes to the variability between co-ES experiments.

**Fig. 6:**
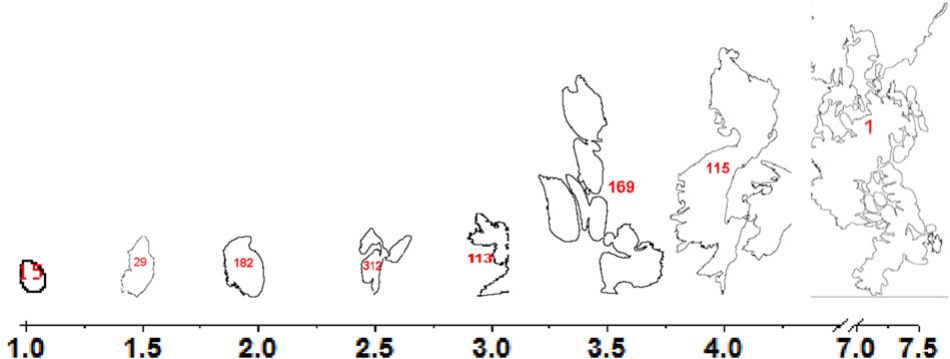
Pore shape parameter *γ* vs. the corresponding pores extracted from the SEM image in Fig. 3a. Note: numbered pores here were for shape demonstration and not scaled, though a large *γ* usually means a large size pore based on the observations of SEM image.

The main goal of pore shape characterization was to determine how the core flow rate and production time affect pore geometry of co-ES fiber bundles. The SEM images of these samples were analyzed according to the described methodology to calculate the pore shape parameter (*γ*). As shown in Fig. 6, the pore geometry became increasingly complex with the increasing value of pore shape parameter. For example, when *γ* ≤ 2.0, the numbered pores for example, no. 15, no. 29 and no. 182 (same pore numbers as in Fig. 3d), could each represent a single pore belonging to either category I or II, depending on pore sizes; when *γ* ≥ 2.5, the pore geometries (no. 312, no. 113 and no. 169) could be formed by a few merging pores in category I or II, or if the boundary among them was not well-defined in the binary images; when *γ* ≥ 4.0, void pores in category III could be included together with pores in category I and/or II forming the complex geometries (no. 115 and no. 1).

**Fig. 7:**
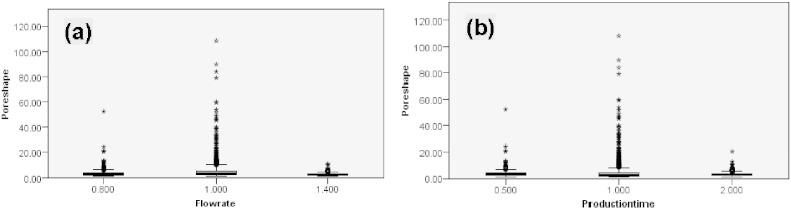
Box-and-whisker plots representing pore shape of fiber bundles produced at various core flow rates and production times. (a) Core flow rate (0.8 mL/h, 1.0 mL/h and 1.4 mL/h); (b) production time (0.5 h, 1.0 h and 2.0 h). Median values, interquartile ranges (i.q.r.) and 1.5 times the i.q.r (excluding outliers ° and extreme values *) are denoted by horizontal bars, boxes and whiskers respectively. The change in pore shape parameter was significant with the increasing core flow rate (Kruskal–Wallis, p < 0.01) but not with production time (Kruskal–Wallis, p = 0.815).

As shown in Fig. 7, there were wide variations in pore shape parameter *γ* with flow rates; but the variation was less with production times. The change in *γ* was significant with flow rate (Kruskal–Wallis, p < 0.01) but not with production time (Kruskal–Wallis, p = 0.815). Outliers and extremes were observed in all fiber bundles, among of which 1.0 mL/h and 1.0 h bundles were more obvious than others, indicating that there were more pores with complex shapes in the binary images, for instance Fig. 4e. Those geometrically complex pores (usually having large sizes) did contribute to the variation in the porosity of fiber bundles, as shown in Table 1. For example, the porosity changed only 6.3% at 1.4 mL/h, while it was 15.9% at 0.8 mL/h or 20.9% at 1.0 mL/h. Spearman's rho correlation further reveals that there is a significant correlation between flow rate and pore shape (rho = − 0.08; p < 0.01, 2-tailed) but not between production time and pore shape (rho = − 0.009; p = 0.654, 2-tailed). The effect of flow rate and production time on pore shape of fiber bundles is totally unpredictable.

It is worth noting that the cross-sections of co-ES bundles are likely to be affected by mechanical cuts perpendicular to the bundles using sharp scissors, which can severely distort the fine structures of co-ES fiber bundles. In most cases, relatively clean cross-sections were obtained. Considerable care was taken to minimize the effect of mechanical cuts on the hollow microfibers. Considering the fact that the freeze fracture method often resulted in elliptical shapes to the pores, there are also inevitable errors with the calculated diameters.

### 3.2 Cross-sectional and longitudinal characterizations of co-ES microfiber strips

During the production of aligned fibers, the accumulating fiber deposition resulted in a building up of the fiber layer on the collector which interrupted the jet stability and thus placed limits on the thickness of the resultant fiber bundles. A method for collecting aligned co-ES microfibers in a strip a few centimeters wide was therefore used to prevent this. This involved the use of a rotating circular drum mounted onto an *x*–*y* translation stage. We found it easier to assemble wider and more uniform structures using these strips, which is an advantage over fiber bundles.

**Fig. 8:**
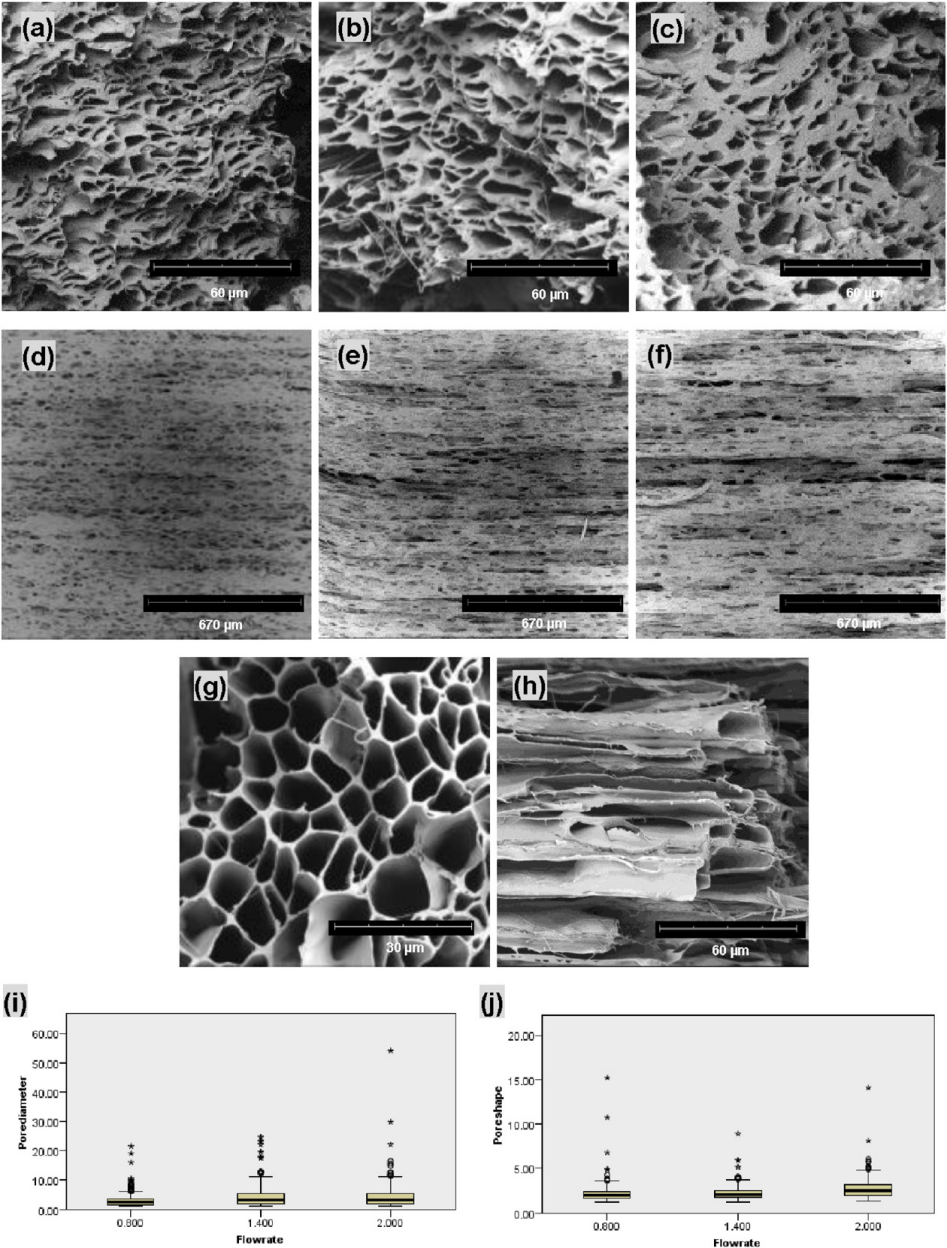
SEM images of (a–c) cross-sections; and (d–f) top surface of three co-ES strips produced at the core flow rate of 0.8 mL/h, 1.4 mL/h and 2.0 mL/h; (g) cross-section of 1.4 mL/h fiber bundle; (h) longitudinal section of 1.4 mL/h fiber strip; (i–j) box-and-whisker plots representing pore size (*d*_*n*_) and pore shape parameter (*γ*) against core flow rate. Median values, interquartile ranges (i.q.r.) and 1.5 times the i.q.r (excluding outliers ° and extreme values *) are denoted by horizontal bars, boxes and whiskers respectively. The changes in pore size and pore shape were significant with the increasing core flow rate (Kruskal–Wallis, p < 0.01).

As shown in Fig. 8a–c, pores and merged fibers were observed on the cross-section of all three fiber strips. In particular, the increase in the core flow rate caused increasingly serious fiber merging, resulting in thick walls between pores as evidenced by Fig. 8c. Fig. 8d–f shows the top surface morphology along the longitudinal direction of three strips. Individual microfibers on the strip top surface were still distinguishable, but were often merged with neighbouring fibers. They also adopted a wavy appearance, while the overall fiber alignment was retained. However, the bottom surface morphology did not show any individual fibers due to a merging into a thin layer of polymer at the point of contact with the collector.

It was also found that the cross-sections were not as well-defined as the porous structure of the fiber bundles, even when the same core flow rate (1.4 mL/h) was used, as seen in Fig. 8b and g. Longitudinal sections (Fig. 8h) further revealed the alignment and fusion between co-ES fibers. Fiber strips made at 0.8 mL/h, 1.4 mL/h and 2.0 mL/h core flow rate displayed significant variations (Kruskal–Wallis, p < 0.01) in pore sizes and pore shape (Fig. 8j–k). However, there was only 1.7% change in porosity when the core flow rate increased from 0.8 mL/h to 1.4 mL/h (52.7% vs 53.6%), but an 18% decrease at 2.0 mL/h (43.9%). Spearman's rho correlation shows that there were very weak correlations between flow rate and pore diameter (rho = 0.141; p < 0.01, 2-tailed) and also between flow rate and pore shape (rho = 0.278; p = 0.654, 2-tailed). Therefore, the effect of flow rate and production time on pore shape of fiber strips is also largely unpredictable.

### 3.3 Effect of *x*–*y* translation speed on cross-sections of fiber strips

**Fig. 9:**
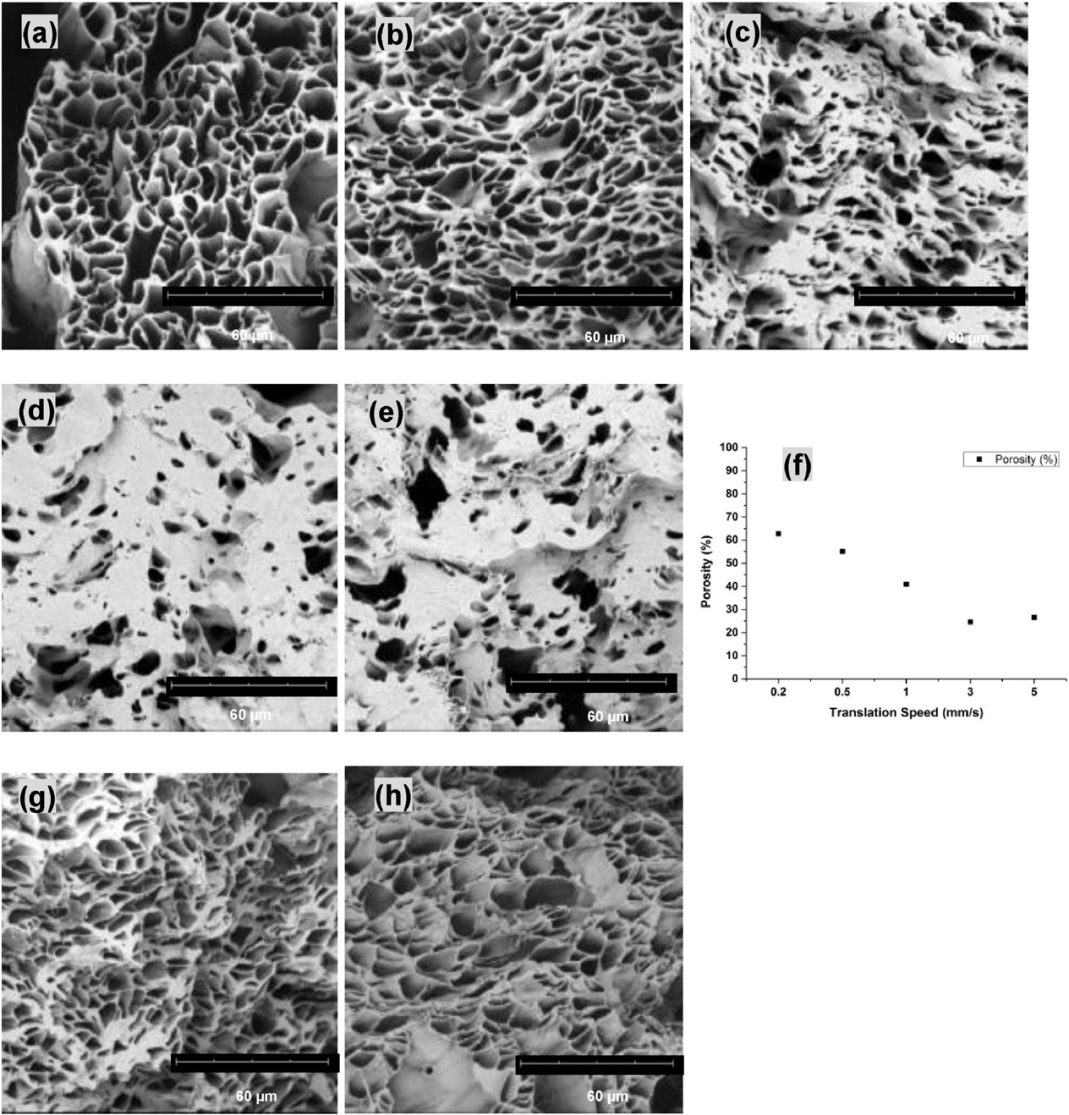
SEM images of co-ES fiber strips using various translation speeds of (a) 0.2 mm/s; (b) 0.5 mm/s; (c) 1 mm/s; (d) 3 mm/s; and (e) 5 mm/s at the core flow rate of 0.8 mL/h; (f) cross-sectional porosity; (g) a 30 mm wide fiber strip produced using 0.5 mm/s and 12 h; (h) a 15 mm wide strip produced using 0.5 mm/s and 6 h.

As mentioned in Section 3.2, co-ES fiber strips were deposited wet on the rotating drum collector and consequently merged with other fibers that had previously been deposited. It is expected that the transition speed may affect fiber deposition and thus the merging of fibers. In order to investigate these effects the translation speed of the *x*–*y* stage was varied from 0.2 mm/s to 5 mm/s during the co-ES process (1 mm/s used in Section 3.2). As shown in Fig. 9a–e, fiber merging occurred in all five strips but became much more pronounced with an increase in the translation speed. Moreover, as demonstrated in Fig. 9f, the cross-sectional porosity firstly decreased from 60% at 0.2 mm/s translation speed (Fig. 9a) to 25% at 3 mm/s (Fig. 9d) and then remained nearly constant at 5 mm/s (Fig. 9e), where the majority of co-ES merged together, leading to the lowest porosity. This is not surprising considering the fact the solvent probably remained in fiber layers deposited early on in the process and had more time to evaporate before a new layer of fibers were deposited on the collector when moving at lower translation speeds. When a low translation speed, for example 0.5 mm/s, was used, thicker fiber strips still having porous cross-sections were produced by increasing the production time from 6 h to 12 h (~ 1.4 mm thick, Fig. 9g) or reducing the strip width from 30 mm to 15 mm (~ 2.2 mm thick, Fig. 9h) while maintaining other co-ES parameters. These fiber strips could be directly used to construct an ultrafiltration membrane module without the use of epoxy glue [10].

### 3.4 Cross-sectional structural reproducibility and variation

**Fig. 10:**
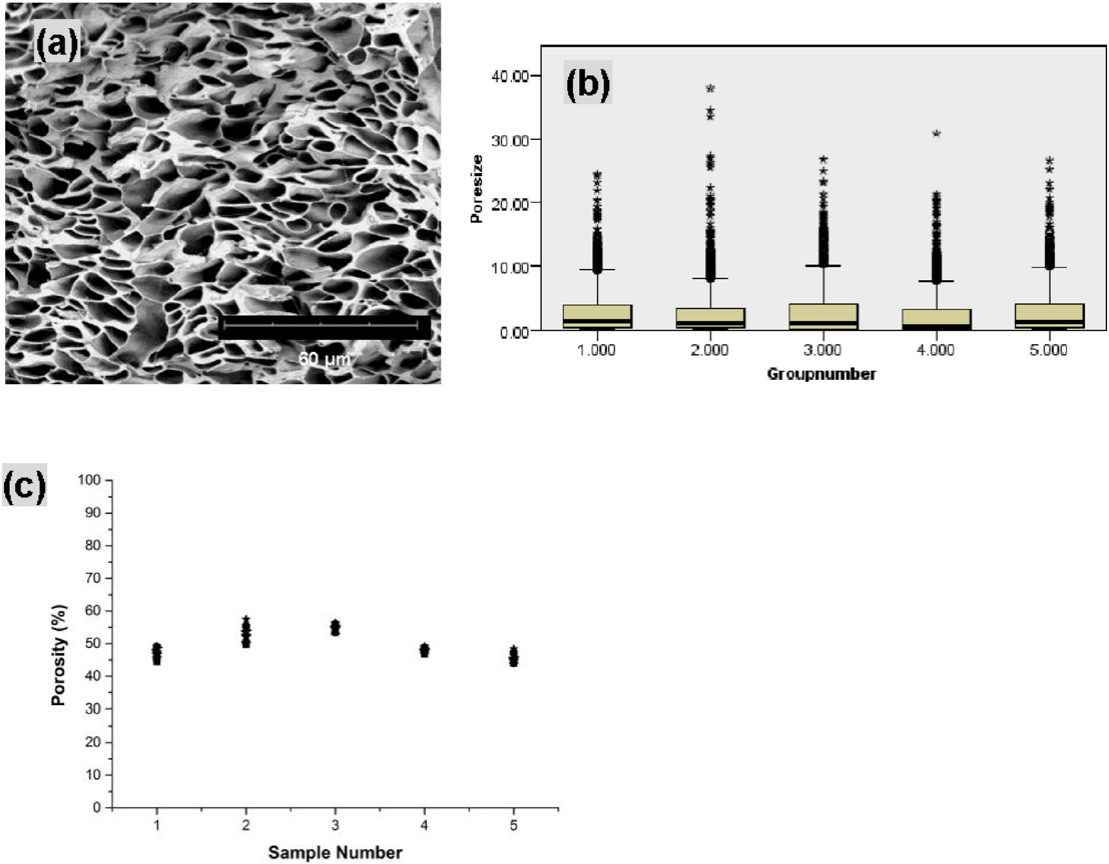
(a) A representative SEM image (2000 × magnification) of a cross-section of a strip created using 0.8 mL/h core flow rate; (b) Box-and-whisker plots representing pore size (*d*_*n*_) of five fiber strips. Median values, interquartile ranges (i.q.r.) and 1.5 times the i.q.r (excluding outliers ° and extreme values *) are denoted by horizontal bars, boxes and whiskers respectively. The variations in pore sizes were small but significant (Kruskal–Wallis, p < 0.01); (c) Plot of porosity for each of five strips.

The reproducibility of co-ES fiber strips was evaluated using 50 SEM images acquired at a 2000 × magnification from five samples (ten images from each) produced using the same experimental conditions (applied voltage 9 kV, working distance 5 cm, shell flow rate 3 mL/h and core flow rate 0.8 mL/h). A representative SEM image of a cross section of a strip is shown in Fig. 10a. Fig. 10b shows small but significant variation in the pore sizes across five groups of fiber strips (Kruskal–Wallis, p < 0.01), which was not entirely surprising considering that fact all pores in categories I, II and III were taken into account. However, no significant changes were seen among the area-weighted pore sizes of these fiber strips (one-way ANOVA, p > 0.05), which was pointed out in our previous study. As shown in Fig. 10c**,** systematic differences of approximately 10% (45.3%–54.4%) were observed in the porosity between the strips, indicating that the reproducibility of porosity was found to be reasonably good.

**Fig. 11:**
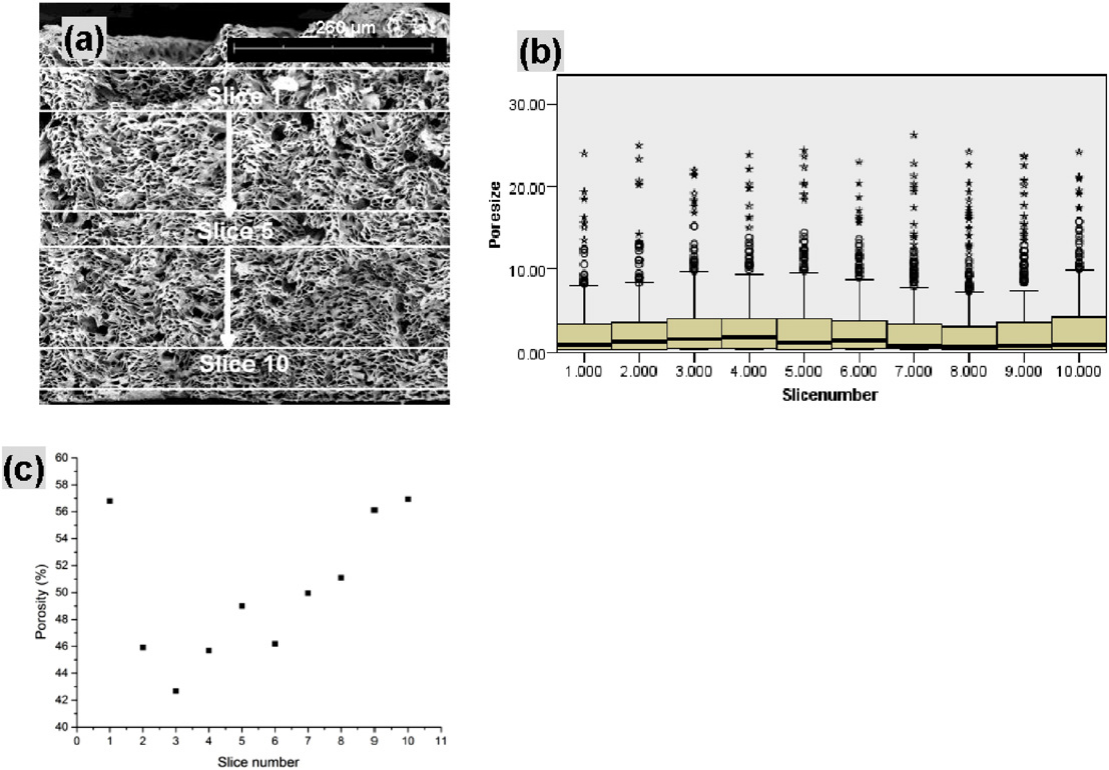
(a) SEM image (500 × magnification) showing whole cross section of a strip and ten non-overlapping sliced areas for porosity measurement (only 3 slices shown, slices not scaled); (b) Box-and-whisker plots representing pore size (*d*_*n*_) against slice number. Median values, interquartile ranges (i.q.r.) and 1.5 times the i.q.r (excluding outliers ° and extreme values *) are denoted by horizontal bars, boxes and whiskers respectively. The change in pore sizes from top to bottom of the strip wasn't significant (Kruskal–Wallis, p > 0.05, pairwise comparisons); (c) plot of porosity (Φ) against slice number from the top to the bottom of a strip.

SEM images (500 × magnification) of the whole cross-section of co-ES strips were also acquired, which was used to observe the variation of pore size and porosity across the sample's thickness. As shown in Fig. 11a, the cross-sectional image was equally divided into 10 spaced regions of interest, where pore size and porosity was measured. There were no significant variations (Kruskal–Wallis, p > 0.05, pairwise comparisons) in pore sizes between any two neighbouring slices from slice 1 to 10 (Fig. 11b). These pores on the top of fiber strip (slice 1) did not differ significantly from those in the middle (slice 5) and bottom (slice 10) of fiber strip. However, as shown in Fig. 11c, with an increasing thickness of fiber strip the porosity across the slices gradually decreased from 56.9% on slice 10 to 42.7% on slice 3, and then increased to 56.8% on slice 1 (adjacent to fiber strip surface), which was almost the same level as the bottom slice (slice 10). This can be understood in terms of rapid evaporation of the core solvent (in the order of tens of seconds [10]) for thin strips and on the strip surface during the collection of PCL microfibers on the rotating drum. That is to say, the reduced core solvent evaporation inside fiber strips can result in a higher packing density of hollow microfibers thus reducing porosity.

## 4 Conclusions

In this work uniaxially aligned hollow microfiber bundles and strips with thicknesses of up to ~ 2 mm were produced by co-ES and characterized by SEM. Porous cross-sectional structures were confirmed by SEM images obtained after freeze fracturing co-ES microfibers. SEM images were processed using ImageJ software tools to obtain cross-sectional pore sizes and porosities of co-ES bundles and strips. The effect of production time, core flow rate and translation speed on the cross-sections of co-ES fibers were studied. The results of Kruskal–Wallis ANOVA tests show that core flow rate had a significant effect on both pore size and pore shape parameter of fiber bundles and strips; however, the effect of production time on pore shape was less significant than fiber shape parameter. Fiber strips, which were controllable in thickness and width, had different cross-sectional structures from fiber bundles, even when produced under similar conditions. It was also found that fiber strips were more prone to merge together with an increasing translation speed of the fiber collector, resulting in a dramatic decrease in the cross-sectional porosity. An optimized low translation speed (i.e. less than 1 mm/s) when co-ES parameters were maintained in the same condition as described in this study allowed us to produce thicker strips still having porous cross sections. Cross-sectional structures of five fiber strips produced in the same experiment settings had reasonably good reproducibility, due to the fact that the amount of variations in pore size and porosity between samples are very small, even if it is statistically significant. For a fiber strip with its cross section equally sectioned into 10 slices, there weren't significant changes in pore sizes between neighbouring slices from top to bottom of the strip; the porosity of these slices decreased with the increasing thickness but returned to a similar value at the slice adjunct to the strip surface. These findings have implications for the process control and product quality of scaled up quantity production of co-ES hollow microfibrous assemblies.

## Notes

The authors declare no competing financial interest.

## Figures and Tables

**Table 1:**
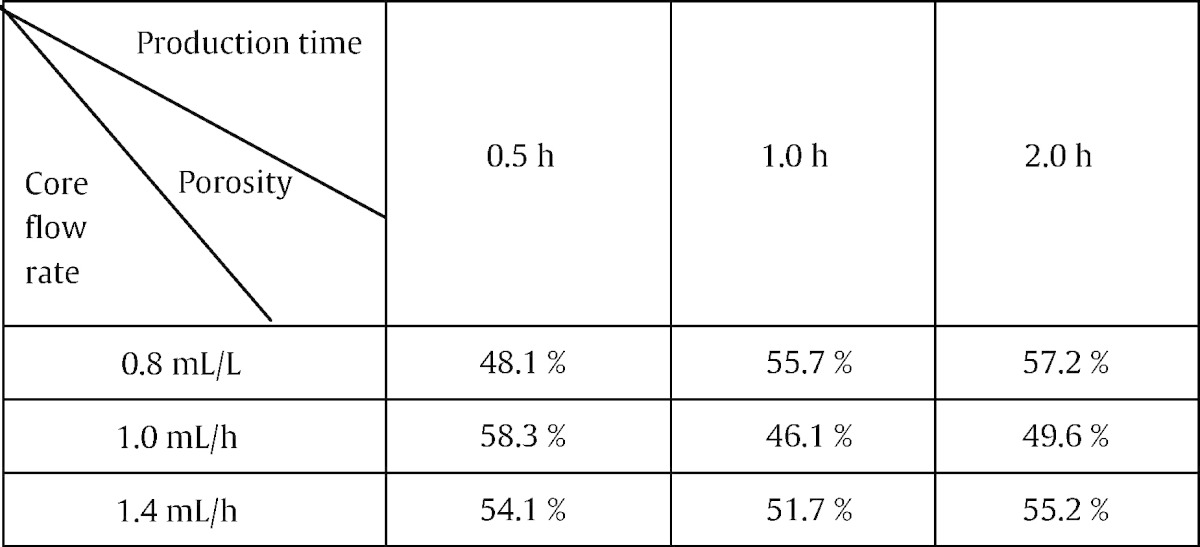
Porosity of fiber bundles.
